# Effects of Early-Stage Blood Pressure Variability on the Functional Outcome in Acute Ischemic Stroke Patients With Symptomatic Intracranial Artery Stenosis or Occlusion Receiving Intravenous Thrombolysis

**DOI:** 10.3389/fneur.2022.823494

**Published:** 2022-03-08

**Authors:** Mian-Xuan Yao, Dong-Hai Qiu, Wei-Cheng Zheng, Jiang-Hao Zhao, Han-Peng Yin, Yong-Lin Liu, Yang-Kun Chen

**Affiliations:** Department of Neurology, Affiliated Dongguan People's Hospital, Southern Medical University, Dongguan, China

**Keywords:** blood pressure variability, symptomatic intracranial artery stenosis or occlusion, intravenous thrombolysis, acute ischemic stroke, prognosis

## Abstract

**Background:**

Studies exploring the relationship between blood pressure (BP) fluctuations and outcome in acute ischemic stroke (AIS) patients treated with intravenous thrombolysis (IVT) are limited. We aimed to investigate the influence of blood pressure variability (BPV) during the first 24 h after IVT on early neurological deterioration (END) and 3-month outcome after IVT in terms of different stroke subtypes.

**Methods:**

Clinical data from consecutive AIS patients who received IVT were retrospectively analyzed. The hourly systolic BP of all patients were recorded during the first 24 h following IVT. We calculated three systolic BPV parameters, including coefficient of variability (CV), standard deviation of mean BP (SD) and successive variation (SV), within the first 6, 12, and 24 h after IVT. END was defined as neurological deterioration with an increase in the National Institutes of Health Stroke Scale (NIHSS) score ≥ 4 points within the first 72 h after admission. Follow-up was performed at 90 days after onset, and favorable and poor outcomes were defined as a modified Rankin Scale scores (mRS) of ≤1 or ≥2, respectively.

**Results:**

A total of 339 patients, which were divided into those with (intracranial artery stenosis or occlusion group, SIASO group) and without (non-SIASO group) SIASO, were included. Among them, 110 patients (32.4%) were with SIASO. Patients in SIASO group had higher NIHSS on admission and difference in term of mRS at 90 days compared with non-SIASO group (*P* < 0.001). In SIASO group, patients in favorable outcome group were younger and had lower NIHSS on admission, lower SV-24 h (14.5 ± 4.3 vs. 11.8 ± 3.2, respectively) and lower SD-24 h (12.7 ± 3.8 vs. 10.9 ± 3.3, respectively), compared with patients with poor outcome (all *P* < 0.05). In the multivariable logistic regression analysis, compared with the lowest SV (SV < 25% quartile), SV_50−75%_ [odds ratio (OR) = 4.449, 95% confidence interval (CI) = 1.231–16.075, *P* = 0.023] and SV_>75%_ (OR = 8.676, 95% CI = 1.892–39.775, *P* = 0.005) were significantly associated with poor outcome at 3 months in patients with SIASO, adjusted for age, NIHSS on admission and atrial fibrillation. No BPV parameters were associated with END in SIASO group. In non-SIASO group, there were no significant association between BPV patterns and END or 90-day outcome.

**Conclusions:**

SV-24 h had a negative relationship with 3-month outcome in AIS patients with SIASO treated with IVT, indicating that BPV may affect the outcome of AIS.

## Background

Globally, stroke is the second most common cause of death and the third most common disabling disease (1, 2). Intravenous thrombolysis therapy (IVT) can significantly improve functional outcomes. However, previous studies have reported that IVT outcomes are associated with various factors (3–5), including age (6), Trial of Org 10172 in Acute Stroke Treatment (TOAST) classification (7–10), National Institutes of Health Stroke Scale (NIHSS) score on admission, and systolic blood pressure (SBP) on admission (11).

Previous studies have confirmed that racial difference in intracranial atherosclerosis stenosis (ICAS), and non-Caucasian population are at higher risk of ICAS (12–16). The incidence of ICAS is up to 46.6% in Chinese with ischemic (17). ICAS is an important risk factor for poor prognosis after acute ischemic stroke (AIS) (18, 19).

Blood pressure variability (BPV) is the fluctuation of blood pressure (BP) over a certain period. BP fluctuations are a process of constant adjustment in response to various environmental factors, both inside and outside the body, to better adapt to the needs of the body. Fluctuations of BP are related to a range of factors, such as mood, temperature, and circadian rhythms. These factors ultimately influence BP by affecting nerve and humoral regulation (20). Recently, higher BPV within 24 h after stroke was reported to be associated with poor outcome after IVT (11, 19, 21–23). However, ICAS, which is identified to be associated with poor outcome after AIS as we mentioned before, was not analyzed in these previous studies. Hemodynamic change is one of the possible mechanisms for cerebral infarction caused by ICAS. The hemodynamic status in the ICAS territory can be easily altered by BP fluctuation (24, 25). Under normal condition, cerebral autoregulation occurs to maintain the cerebral blood flow against the effect of hypoperfusion or hyperperfusion (26). However, cerebral autoregulation is impaired after ischemic stroke, especially for patients with ICAS (24). The fluctuation of peripheral blood pressure can significantly affect cerebral perfusion pressure in these patients. A rapid drop of BP may easily lead to a rapidly decreasing perfusion, especially in patients with ICAS, while a rapid rise of BP may potentially lead to intracranial hemorrhage, which might result in early neurological deterioration (END) and poorer outcome (24, 25). Nevertheless, studies focusing on the relationship between BPV and outcome of AIS patients with symptomatic ICAS are still limited. In the present study, we aimed to explore the influence of BPV during the first 24 h after IVT on END and 3-month outcome in patients who had intracranial artery stenosis or occlusion (SIASO) and received IVT.

## Methods

### Patient Selection

Patients with AIS who were treated with recombinant tissue plasminogen activator (r-tPA) IVT and admitted to Dongguan People's Hospital between 1 January 2017 and 31 December 2019 were consecutively recruited. The inclusion criteria were as follows: (1) aged >18 years; (2) AIS was confirmed by magnetic resonance imaging (MRI) during hospitalization; (3) onset of ischemic stroke symptoms was within 4.5 h of treatment with r-tPA; (4) clinical features and BP were recorded at baseline and then hourly for 24 h after IVT; (5) prestroke modified Rankin Scale (mRS) score ≤1; and (6) follow-up by face-to-face consultation or by phone at 3 months, with complete documentation. The exclusion criteria were as follows: (1) additional endovascular therapy after IVT; (2) incomplete clinical data or lost to follow-up. This study was approved by the ethics committee of Dongguan People's Hospital (approval number: KYKT2018-002). The study was considered exempt from requiring informed consent from patients because of its retrospective nature.

### Definition of SIASO

SIASO was defined as no <50% stenosis of the intracranial artery that was responsible for the AIS (27). Symptomatic intracranial artery occlusion (SIAO) was defined as signal loss of distal blood flow ipsilateral to the infarction (27). Patients who didn't meet the criteria of SIAO were those with symptomatic intracranial artery stenosis (SIAS) in SIASO group. The degree of stenosis was measured using three-dimensional time-of-flight magnetic resonance angiography [the MRI parameters are detailed in our previous article (28)] by comparing the vessel diameter at the site of stenosis with the normal vessel diameter distal to the stenosis (29).

### Data Collection

Demographic data including age, sex, and a history of hypertension, diabetes mellitus, smoking, atrial fibrillation (AF), and previous stroke were collected. Stroke subtypes were classified according to the TOAST criteria (30), and the NIHSS on admission, onset-to-treatment time, SBP on admission, and hourly SBP for 24 h after IVT were also collected.

### Definition of BPV

The hourly SBPs of all patients were recorded during the first 24 h after IVT using a cuff-type BP monitor. According to the guidelines (31), intravenous urapidil was administered to reduce BP levels to <185/110 mmHg just before r-tPA administration, and to maintain BP levels at <180/105 mmHg during the first 24 h after IVT. The BPV was calculated using the following equation (19):

(1) Standard deviation of mean BP (SD): $\sqrt{1/\left(n-1\right)\overset{\left(n-1\right)}{\underset{i=1}{\sum }}\left(BP_{i}-BP_{mean}\right)}$,
(2) Coefficient of variability (CV [%]): SD/BP_mean_ × 100,
(3) Successive variation (SV): $\sqrt{1/\left(n-1\right)\overset{\left(n-1\right)}{\underset{i=1}{\sum }}{\left(BP_{i+1}-BP_{i}\right)}^{2}}$ (19).

### Definition of END

In our study, the definition of END referred to neurological deterioration, with an increase in NIHSS score ≥4 points in the first 72 h after admission (32).

### Clinical Outcome

We followed up these patients and assessed their mRS scores at 90 days after onset. Favorable and poor outcomes were defined as mRS ≤1 and ≥2, respectively. Incidents of stroke recurrence and death within the follow-up period were also recorded.

### Analysis of BPV

BPVs within 6 h (SV-6 h, SD-6 h, CV-6 h), 12 h (SV-12 h, SD-12 h, CV-12 h), and 24 h (SV-24 h, SD-24 h, CV-24 h) after IVT was calculated.SV was a continuous variable and SV-24 h was divided into four grades based on the quartiles. We assessed the relationship between SV and END, so did the relationship between SV and the 3-month outcome. We also assessed the relationship between each interval of SV-24 h and END, as well as the relationship between each interval of SV-24 h and the 3-month outcome.

### Statistical Analysis

Statistical analyses were conducted using SPSS for Windows (v.20.0; IBM Corp., Armonk, NY, USA). Continuous variables with a normal distribution were reported as the mean ± SD and non-normally distributed variables as the median and interquartile range. Univariate analyses were performed using the *t*-test/Mann–Whitney *U*-test (for two groups) or one-way analysis of variance/Kruskal–Wallis *H*-test (for three or more groups) for continuous variables, and the χ^2^ test for categorical variables. Variables with *P* < 0.05 in the univariate analysis were included in further binary multivariate logistic regressions. Statistical significance was defined as *P* < 0.05 (two-sided). Statistical graphs were drawn using GraphPad Prism (v.9.0; GraphPad Software, San Diego, CA, USA).

## Results

### Patients' Characteristics

**Figure 1:**
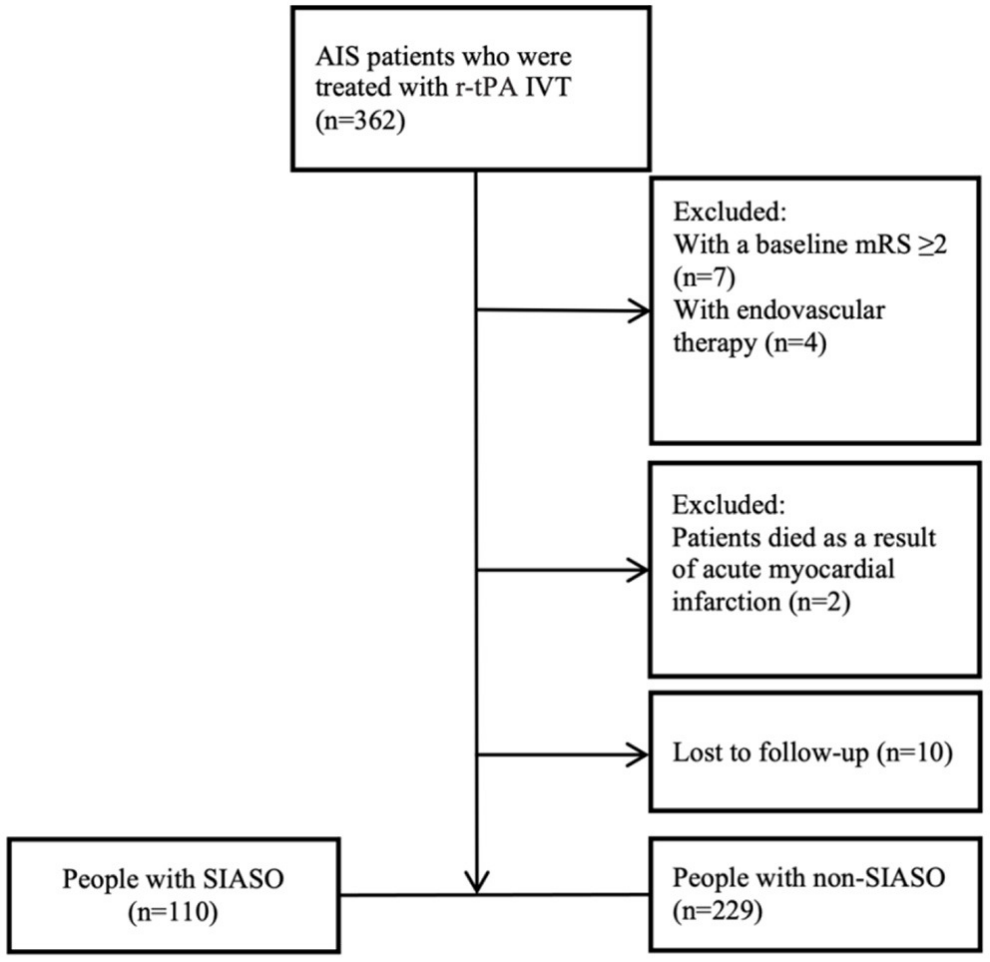
Flowchart of the selection process. AIS, acute ischemic stroke; IVT, intravenous thrombolysis; mRS, modified rankin scale; SIASO, symptomatic intracranial artery stenosis or occlusion.

Three hundred and sixty-two AIS patients who were treated with r-tPA IVT in our stroke unit were collected consecutively. Among these 362 patients, 23 patients were excluded for the following reasons: baseline mRS ≥ 2 (*n* = 7), underwent additional endovascular therapy (*n* = 4), died as a result of acute myocardial infarction (*n* = 2), and lost to follow-up (*n* = 10). Therefore, 339 patients were enrolled in this study and among them, 110 patients were with SIASO (Figure 1).

These 339 patients had a mean age of 61.9 ± 12.1 years. Among them, 248 patients (73.2%) were male, and urapidil was used to lower BP in 34 patients (10.0%) before IVT. Forty-two patients (12.4%) had END and 195 patients (57.5%) had a poor outcome at the 3-month follow-up (Table 1).

**Table 1:** Characteristics of the study sample.

**Characteristics**	**Total sample (*n* = 339)**	**SIASO group (*n* = 110)**	**Non-SIASO group (*n* = 229)**	***P*-value**
Age^a^ (years)	61.9 (12.1)	62.0 (12.5)	61.8 (12.0)	0.864
Men^b^, *n* (%)	248 (73.2%)	86 (78.2%)	162 (70.7%)	0.148
Hypertension^b^, *n* (%)	239 (70.5%)	73 (66.4%)	166 (72.5%)	0.247
Use antihypertensive therapy before IVT^b^, *n* (%)	34 (10.0%)	9 (8.2%)	25 (10.9%)	0.330
Diabetes mellitus^b^, *n* (%)	91 (26.8%)	29 (26.4%)	62 (27.1%)	0.890
History of hypercholesterolemia^b^, *n* (%)	132 (38.9%)	41 (37.3%)	91 (39.7%)	0.806
Smokers/ex-smokers^b^, *n* (%)	126 (37.2%)	48 (43.6%)	78 (34.1%)	0.088
AF^b^, *n* (%)	73 (21.5%)	23 (20.9%)	50 (21.8%)	0.846
History of coronary heart disease^b^, *n* (%)	18 (5.3%)	9 (8.2%)	19 (8.3%)	0.971
Previous stroke^b^, *n* (%)	56 (16.5%)	23 (20.9%)	33 (14.4%)	0.131
Systolic blood pressure on admission^a^	156.5 (24.7)	153.7 (24.0)	158.3 (25.0)	0.141
OTT^a^ (minutes)	195.2 (55.9)	194.2 (54.8)	195.7 (56.6)	0.823
NIHSS on admission^c^ (IQR, 25–75)	7.0 (4.0–10.0)	9.0 (5.8–14.0)	6.0 (4.0–9.0)	**<0.001**
SIAS^b^, *n* (%)	84 (24.8%)	84 (76.4%)	–	–
SIAO^b^, *n* (%)	26 (7.7%)	26 (23.6%)	–	–
END^b^, *n* (%)	42 (12.4%)	20 (18.2%)	22 (9.6%)	0.019
HT^b^, *n* (%)	74 (21.8%)	33 (30.0%)	41 (17.9%)	**0.012**
sHT^b^, *n* (%)	10 (2.9%)	6 (5.5%)	5 (2.2%)	0.112
mRS at 90 days^b^, *n* (%)				**<0.001**
0	121 (35.7%)	20 (18.2%)	101 (44.1%)	
1	74 (21.8%)	17 (15.5%)	57 (24.9%)	
2	49 (14.5%)	21 (19.1%)	28 (12.2%)	
3	41 (12.1%)	17 (15.5%)	24 (10.5%)	
4	31 (9.1%)	19 (17.3%)	12 (5.2%)	
5	10 (2.9%)	6 (5.5%)	4 (1.7%)	
6	13 (3.8%)	10 (9.1%)	3 (1.3%)	

a *Mean (SD), t-test*.

b *n (%), chi-square test*.

c *Mann–Whiteny U-test*.

*NIHSS, national institutes of health stroke scale; OTT, onset to treatment time; AF, atrial fibrillation; mRS, modified rankin scale; END, early neurological deterioration; IVT, intravenous thrombolysis; SIASO, symptomatic intracranial artery stenosis or occlusion; SIAS, symptomatic intracranial arterial stenosis; SIAO, symptomatic intracranial arterial occlusion; HT, hemorrhagic transformation; sHT, symptomatic hemorrhagic transformation. The bold values mean P < 0.05*.

We divided these 339 patients into two groups (SIASO group and non-SIASO group) based on whether they were with SIASO. There is no difference between these two groups in terms of age (62.0 ± 12.5 vs. 61.8 ± 12.0 years, *P* = 0.864), male (78.2 vs. 70.7%, *P* = 0.148), hypertension (66.4 vs. 72.5%, *P* = 0.247), diabetes mellitus (26.4 vs. 27.1%, *P* = 0.890), smokers/ex-smokers (43.6 vs. 34.1%, *P* = 0.088), AF (20.9 vs. 21.8%, *P* = 0.846), previous stroke (20.9 vs. 14.4%, *P* = 0.131), onset to treatment time (OTT, 194.2 ± 54.8 vs. 195.7 ± 56.6 min, *P* = 0.823) or symptomatic hemorrhagic transformation (sHT, 5.5 vs. 2.2%, *P* = 0.112). Patients in the SIASO group had a higher NIHSS on admission [9.0 (5.8–14.0) vs. 6.0 (4.0–9.0), respectively], a higher mRS at 90 days and a higher rate of hemorrhagic transformation (HT) compared with the non-SIASO group (*P* < 0.05, respectively). In the SIASO group, there were 26 patients (23.6%) with SIAS and 84 patients (76.4%) with SIAO. The respective characteristics of patients are shown in Table 1.

In SIASO group, there were significant differences among these four groups of SV quartiles in terms of age, hypertension, use of antihypertensive therapy before IVT, AF, and mRS score at 90 days. In the non-SIASO group, there were significant differences among the quartiles of SV in terms of hypertension, smokers/ex-smokers, systolic blood pressure on admission, OTT and NIHSS on admission (Table 2). In the univariable analysis of risk factors for END, there were no differences in age, AF, SBP on admission, OTT, NIHSS on admission, or medical history between the SIASO and non-SIASO groups (Table 3; Supplementary Table 1).

**Table 2:** Successive blood pressure variability of the study sample.

**Characteristics**	**SIASO group (*****n*** **=** **110)**					**Non-SIASO (*****n*** **=** **229)**				
	**SV_ <25%_ <10.84 (*n* = 27)**	**SV_**25−50*%***_ = 10.84–13.25 (*n* = 28)**	**SV_**50−75*%***_ = 13.26–16.00 (*n* = 27)**	**SV_**>75%**_ > 16.00 (*n* = 28)**	***P*-value**	**SV_ <25%_ <10.08 (*n* = 57)**	**SV_25−50*%*_ = 10.08–12.93 (*n* = 57)**	**SV_50−75*%*_ = 12.94–15.32 (*n* = 58)**	**SV_>75%_> 15.32 (*n* = 57)**	***P*-value**
Age^a^ (years)	55.4 (13.4)	60.2 (14.1)	67.7 (8.7)	64.7 (9.8)	**0.004**	61.4 (12.3)	60.9 (11.6)	61.7 (12.0)	63.2 (12.2)	0.645
Men^b^, *n* (%)	21 (77.8%)	23 (82.1%)	22 (81.5%)	20 (71.4%)	0.475	43 (75.4%)	40 (70.2%)	37 (63.8%)	42 (73.7%)	0.464
Hypertension^b^, *n* (%)	16 (59.3%)	16 (57.1%)	17 (63.0%)	24 (85.7%)	**0.026**	41 (71.9%)	38 (66.7%)	38 (65.5%)	49 (86.0%)	**0.050**
Use antihypertensive therapy before IVT^b^, *n* (%)	1 (3.7%)	1 (3.6%)	0	7 (25.0%)	**0.002**	4 (7.0%)	5 (8.8%)	12 (20.7%)	4 (7.0%)	0.089
Diabetes mellitus^b^, *n* (%)	4 (14.8%)	6 (21.4%)	9 (33.3%)	10 (35.7%)	0.472	12 (21.1%)	16 (28.1%)	17 (29.3%)	17 (29.8%)	0.822
History of hypercholesterolemia^b^, *n* (%)	7 (25.9%)	11 (39.3%)	11 (40.7%)	12 (42.9%)	0.787	19 (33.3%)	23 (40.4%)	22 (37.9%)	27 (47.4%)	0.408
Smokers/ex-smokers^b^, *n* (%)	11 (40.7%)	13 (46.4%)	9 (33.3%)	15 (53.6%)	0.082	24 (42.1%)	11 (19.3%)	21 (36.2%)	22 (38.6%)	**0.042**
AF^b^, *n* (%)	3 (11.1%)	5 (17.9%)	6 (22.2%)	9 (32.1%)	**0.006**	15 (26.3%)	10 (17.5%)	14 (24.1%)	11 (19.3%)	0.575
History of coronary heart disease^b^, *n* (%)	1 (3.7%)	1 (3.6%)	5 (18.5%)	2 (7.1%)	0.705	7 (12.3%)	4 (7.0%)	3 (5.2%)	5 (8.8%)	0.591
Previous stroke^b^, *n* (%)	3 (11.1%)	4 (14.3%)	4 (14.8%)	12 (42.9%)	0.137	9 (15.8%)	7 (12.3%)	10 (17.2%)	7 (12.3%)	0.818
Systolic blood pressure on admission^a^	148.1 (23.8)	150.8 (27.8)	147.1 (21.8)	168.9 (16.1)	0.353	149.3 (23.5)	154.7 (24.7)	167.1 (25.7)	162.1 (22.4)	**0.001**
OTT^a^ (minutes)	183.0 (55.2)	181.1 (52.7)	215.9 (47.8)	197.3 (58.4)	0.067	219.5 (47.9)	185.6 (59.0)	184.0 (59.6)	193.9 (53.1)	**0.011**
NIHSS on admission^c^ (IQR, 25–75)	8.0 (4.0–14.0)	9.5 (6.0–14.0)	7.0 (4.0–15.0)	9.0 (6.3–13.8)	0.091	6.0 (4.0–10.0)	7.0 (4.0–11.0)	5.5 (4.0–8.0)	5.0 (3.0–7.0)	**0.021**
END^b^, *n* (%)	2 (7.4%)	5 (17.9%)	4 (14.8%)	9 (32.1%)	0.320	5 (8.8%)	8 (14.0%)	6 (10.3%)	3 (5.3%)	0.449
mRS at 90 days^b^, *n* (%)					**0.007**					0.232
0	7 (25.9%)	4 (14.3%)	4 (14.8%)	5 (17.9%)		26 (45.6%)	18 (31.6%)	28 (48.3%)	29 (50.9%)	
1	6 (22.2%)	7 (25.0%)	2 (7.4%)	2 (7.1%)		14 (24.6%)	15 (26.3%)	12 (20.7%)	16 (28.1%)	
2	6 (22.2%)	1 (3.6%)	7 (25.9%)	7 (25.0%)		9 (15.8%)	7 (12.3%)	8 (13.8%)	4 (7.0%)	
3	3 (11.1%)	6 (21.4%)	4 (14.8%)	4 (14.3%)		2 (3.5%)	12 (21.1%)	5 (8.6%)	5 (8.8%)	
4	5 (18.5%)	6 (21.4%)	7 (25.9%)	1 (3.6%)		3 (5.3%)	5 (8.0%)	2 (3.4%)	2 (3.5%)	
5	0	2 (7.1%)	2 (7.4%)	2 (7.1%)		2 (3.5%)	0	2 (3.4%)	0	
6	0	2 (7.1%)	1 (3.7%)	7 (25.0%)		1 (1.8%)	0	1 (1.7%)	1 (1.8%)	

a *One-way ANOVA*.

b *x^2^ test*.

c *Kruskal-Wallis H test*.

*SV, successive variability of systolic blood pressure; NIHSS, national institutes of health stroke scale; OTT, onset to treatment time; AF, atrial fibrillation; mRS, modified rankin scale; END, early neurological deterioration; IVT, intravenous thrombolysis; SIASO, symptomatic intracranial artery stenosis or occlusion. The bold values mean P < 0.05*.

**Table 3:** Risk factors of the 3-month poor outcome and END in univariable analysis of SIASO group.

	**Poor outcome (mRS = 2–6) (*n* = 73)**	**Favorable outcome (mRS = 0–1) (*n* = 37)**	***t/X*^2^/*z* value**	***P-*value**	**With END (*n* = 20)**	**Without END (*n* = 90)**	***t/X*^2^/*z* Value**	***P-*value**
Age^a^ (years)	64.8 (10.9)	56.7 (13.8)	−3.129	**0.003**	64.8 (10.3)	61.43 (12.9)	−1.077	0.284
MenTN8, *n* (%)	12 (16.4%)	12 (32.4%)	3.682	0.055	17 (85.0%)	69 (76.7%)	0.666	0.556
HypertensionTN8, *n* (%)	51 (69.9%)	22 (59.5%)	1.191	0.275	17 (85.0%)	56 (62.2%)	3.803	0.067
Use antihypertensive therapy before IVTTN8, *n* (%)	7 (9.6%)	2 (5.4%)	0.572	0.715	4 (20.0%)	5 (5.6%)	4.545	0.055
Diabetes mellitusTN8, *n* (%)	21 (28.8%)	8 (21.6%)	0.646	0.422	6 (30.0%)	23 (25.6%)	0.167	0.683
History of hypercholesterolemiaTN8, *n* (%)	25 (34.7%)	16 (43.2%)	0.756	0.385	6 (68.4%)	35 (61.1%)	0.357	0.550
Smokers/ex-smokersTN8, *n* (%)	31 (42.5%)	17 (45.9%)	0.121	0.728	11 (55.0%)	37 (41.1%)	1.283	0.257
AFTN8, *n* (%)	20 (27.4%)	3 (8.1%)	5.525	**0.024**	6 (30.0%)	17 (18.9%)	1.222	0.269
History of coronary heart diseaseTN8, *n* (%)	8 (11.0%)	1 (2.7%)	2.228	0.268	2 (10.0%)	7 (7.8%)	0.108	0.666
Previous strokeTN8, *n* (%)	19 (26.1%)	4 (10.8%)	3.438	0.083	6 (30.0%)	17 (18.9%)	1.222	0.269
Systolic blood pressure on admission^a^	153.7 (24.4)	153.2 (24.2)	−0.108	0.914	161.2 (25.6)	151.9 (23.7)	−1.488	0.140
OTT^a^ (minutes)	201 (49.8)	180.9 (62.0)	−1.842	0.068	193.0 (56.8)	194.5 (54.6)	0.115	0.909
NIHSS on admission^c^ (IQR, 25–75)	11.0 (7.0–16.0)	6.0 (3.5–9.0)	−4.377	**<0.001**	10 (5.3–16.3)	9 (5.8–14.0)	−0.641	0.522
SV-24 h^a^	14.5 (4.3)	11.8 (3.2)	−3.650	**<0.001**	15.2 (4.3)	13.2 (4.0)	−1.972	0.051
SD-24 h^a^	12.7 (3.8)	10.9 (3.3)	−2.569	**0.012**	13.7 (4.8)	11.7 (3.4)	−1.751	0.093
CV-24 h^a^	8.9 (2.6)	8.0 (2.4)	−3.650	0.092	9.2 (3.2)	8.5 (2.4)	−1.165	0.247

a *Mean (SD), t-test*.

b *n (%), chi-square test*.

c *Mann–Whiteny U-test*.

*SV-24 h, successive variability of systolic blood pressure within the first 24 hours after IVT; SD-24 h, standard deviation of systolic blood pressure within the first 24 hours after IVT; CV-24 h, coefficient of systolic blood pressure variation within the first 24 hours after IVT; NIHSS, national institutes of health stroke scale; OTT, onset to treatment time; IVT, intravenous thrombolysis; AF, atrial fibrillation; mRS, modified rankin scale; END, early neurological deterioration; SIASO, symptomatic intracranial artery stenosis or occlusion. The bold values mean P < 0.05*.

In SIASO group, compared with those with favorable outcome, patients with poor outcome were older, with higher NIHSS scores on admission and were more likely to have AF (Table 3). In non-SIASO group, those with favorable outcome were older, with higher NIHSS scores on admission as well as longer OTT and were more likely to have AF compared with people in poor outcome group (Supplementary Table 1).

### Association Between BPV Parameters and Outcomes

#### Relationship Between BPV Parameters and 3-Month Outcome

Compared with patients in the favorable outcome group, SV-24 h and SD-24 h were significantly higher in the poor outcome group (all *P* < 0.05) in SIASO group, while CV-24 h did not differ between the two groups (Table 3). However, SV-24 h, SD-24 h, and CV-24 h were not related with 3-month poor outcome in non-SIASO group (Supplementary Table 1).

**Figure 2:**
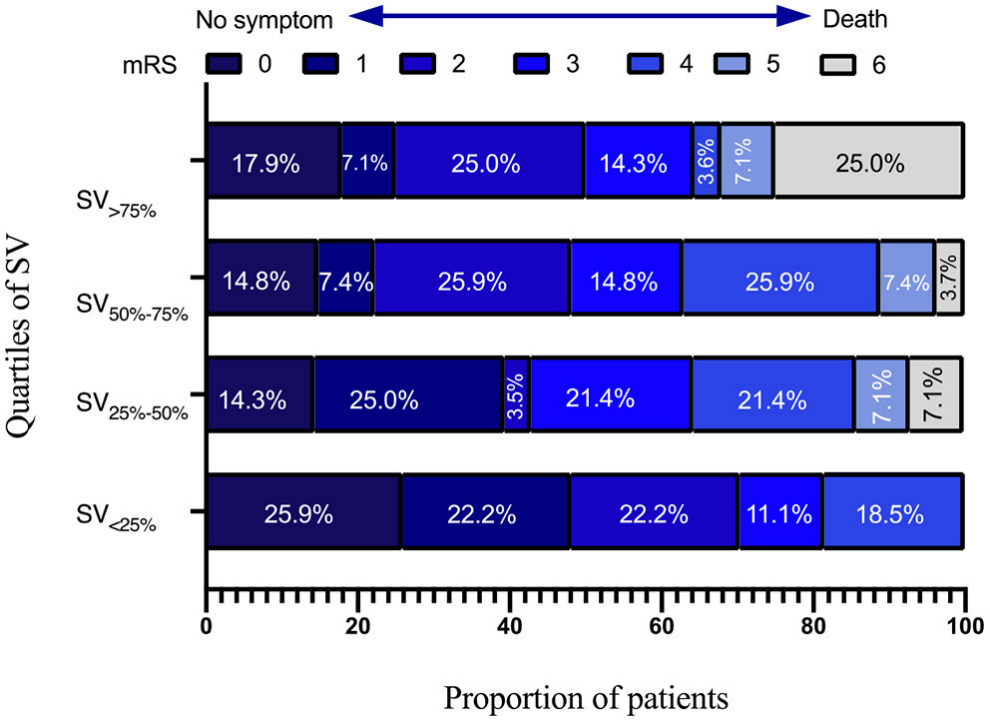
Modified Rankin Scale scores distribution in SIASO group according to quartiles of systolic SV-24 h.

Independent variables in SIASO group that were significantly different between the favorable and poor outcome groups in the univariable analysis (*p* < 0.05) were then entered into the subsequent logistic regression models (Table 4). We set two models for logistic regression. In Model 1, age, NIHSS score on admission, AF, SD-24 h, and overall SV-24 h were entered using a backward Wald method. NIHSS score on admission [odds ratio (OR) = 1.206, 95% confidence interval (CI) = 1.084–1.342, *P* = 0.001] and overall SV (OR = 1.182, 95% CI = 1.035–1.348, *P* = 0.013) were significantly associated with poor outcome at 3 months. In Model 2, patients were divided into four groups based on systolic SV value quartiles (SV_ <25%_ <10.84, SV_25−50%_ = 10.84–13.25, SV_50−75%_ = 13.26–16.00 and SV_>75%_ > 16.00), and these were entered into the model along with the other variables used in Model 1. NIHSS score on admission (OR = 1.227, 95% CI = 1.099–1.369, *P* < 0.001), SV_50−75%_ (SV_ <25%_ as reference; OR = 4.449, 95% CI = 1.231–16.075, *P* = 0.023), and SV_>75%_ (SV_ <25%_ as reference; OR = 8.676, 95% CI = 1.892–39.775, *P* = 0.005) were significant predictors of poor outcome at 3 months (Table 4). The univariable analysis and logistic regression for non-SIASO patients were shown in Supplementary Table 2. The mRS score distribution in SIASO group according to SV quartiles is shown in Figure 2.

**Table 4:** Multivariate logistic regression of risk factors for 3-month poor outcome in SAISO group.

**Variable**	**3-month**		
	**β**	**OR (95% CI)**	***P-*** **value**
**Model^a^**			
Age	0.038	1.038 (0.998–1.081)	0.064
NIHSS on admission	0.187	1.206 (1.084–1.342)	**0.001**
AF	0.204	1.227 (0.278–5.403)	0.787
SV-24 h	0.167	1.182 (1.035–1.348)	**0.013**
SD-24 h	−0.048	0.954 (0.780–1.166)	0.643
**Model^b^**			
Age	0.033	1.033 (0.992–1.076)	0.114
NIHSS on admission	0.204	1.227 (1.099–1.369)	**<0.001**
AF	−0.007	0.993 (0.218–4.520)	0.993
SD-24 h	0.005	1.005 (0.855–1.181)	0.951
SV (SV_ <25%_ as reference)			**0.013**
SV_25−50%_	0.766	2.152 (0.650–7.128)	0.210
SV_50−75%_	1.493	4.449 (1.231–16.075)	**0.023**
SV_>75%_	2.161	8.676 (1.892–39.775)	**0.005**

a *With overall SV entered*.

b *With categorized SV entered*.

*SV-24 h, successive variability of systolic blood pressure within the first 24 hours after IVT; SD-24 h, standard deviation of systolic blood pressure within the first 24 hours after IVT; SIASO, symptomatic intracranial artery stenosis or occlusion; AF, atrial fibrillation. The bold values mean *P* < 0.05*.

#### Interaction Analysis

The interaction analysis of age, AF, hypertension and SV-24 h showed only age and SV-24 h (OR = 1.161, 95% CI = 1.031–1.308, *P* = 0.014) were significantly associated with poor outcome at 3 months in SIASO group, while SV-24 h by age (OR = 0.996, 95% CI = 0.986–1.006, *P* = 0.389), SV-24 h by AF (OR = 1.059, 95% CI = 0.959–1.168, *P* = 0.257) or SV-24 h by hypertension (OR = 1.011, 95% CI = 0.941–1.087, *P* = 0.760) was not significantly associated with poor outcome at 3 months in SIASO group (Supplementary Table 3). Interaction analysis of BPV and SIASO on the 3-month poor outcome in all patients with IVT showed NIHSS on admission and SV-24 h by SIASO (interaction variable between SV-24 h and SIASO) (OR = 1.104, 95% CI = 1.060–1.149, *P* = <0.001), rather than SV-24 h (OR = 0.992, 95% CI = 0.937–1.050, *P* = <0.774) or SIASO (OR = 0.388, 95% CI = 0.067–2.247, *P* = 0.291), were significantly associated with poor outcome at 3 months (Supplementary Table 4).

#### Relationship Between BPV Parameters Within the First 6, 12 h After IVT and 3-Month Outcome

In the univariate analysis, compared with patients in the favorable outcome group, SV-6 h, SV-12 h, and SD-12 h were significantly higher in the poor outcome group (all *P* < 0.05) in SIASO group, while SD-6 h, CV-6 h, and CV-12 h did not differ between the two groups (Supplementary Table 5). Age, NIHSS score on admission, AF, SV-6 h, SD-12 h, and SV-12 h were entered into the subsequent logistic regression models (Supplementary Table 6). SV-6 h (*P* = 0.130), SD-12 h (*P* = 0.960), and SV-12 h (*P* = 0.066) were not significantly associated with poor outcome at 3 months (Supplementary Table 6).

#### Relationship Between BPV Parameters and END

In SIASO group, although SV-24 h, SD-24 h, and CV-24 h all appeared to be higher in END group than in without-END group, there were no significant differences between the two groups (Table 3). In non-SIASO group, SV-24 h, SD-24 h, and CV-24 h were also not significantly different between END and without-END groups (Supplementary Table 1).

#### Relationship Between BPV Parameters and HT or sHT

No relationship was found between the 3 BPV patterns (SV-24 h, SD-24 h, and CV-24 h) and all HT or sHT neither in SIASO group or in non-SIASO group, (Supplementary Tables 7, 8).

## Discussion

In the present study, higher systolic SV-24 was associated with poorer 3-month outcome in patients with SIASO who were treated with IVT. An increase in systolic SV-24 h may thus predict a higher risk of poor outcome at 3 months. However, there was no clear relationship between any pattern of BPV and 3-month outcome in patients without SIASO. There was not relationship between BPV and END in neither of the two groups. Our findings may help physicians to identify patients with a relatively high risk of poor outcome at 3 months.

SV is an important index of BPV, and in our study SV-24 h was associated with 3-month outcome in AIS patients after IVT in patients with SIASO, while it was not associated with 3-month outcome in patients without SIASO. A study by Yong and Kaste (33) in the European Cooperative Acute Stroke Study II trial revealed that high SBP variability is associated with poor outcome. Furthermore, an analysis from the Third International Stroke Trial (IST-3) reported an association between higher BPV and adverse events, the occurrence of symptomatic intracranial hemorrhage, and poor 6-month outcome (34). However, patients with and without SIASO were not assessed separately in those studies. In the IST-3 study, BP was measured only at three timepoints after IVT (34), while we measured BP hourly for at least 24 h after IVT. Thus, the BPV in our study was likely more accurate than in other studies that measured BP less frequently. Additionally, we studied the effect of BPV within different times after IVT on 3 months poor outcome and we found that SV-24 h, rather than BPV patterns within the first 6 or 12 h after IVT, was associated with poor outcomes at 3 months, which was rarely reported in previous studies. Therefore, it is important to avoid excessive fluctuations in BP within 24 h after IVT in patients with SIASO. We also analyzed the 3-month poor outcome of all IVT patients by the interaction of SV-24 h and SIASO, and it showed that the interaction did have a significant effect on 3 months poor outcome, which also confirms the finding that SV-24 h affect the 3-month outcome only in patients with SIASO.

Our study indicated that higher SV-24 h was associated with worse outcome at 3 months after IVT in patients with SIASO rather than in those without SIASO. Although there were some studies on the relationship between BPV and prognosis of AIS after IVT, they did not focus on the patients with SIASO. As ischemic stroke is a clinical syndrome with various pathogenesis, including large artery atherosclerosis, small arterial occlusion, and cardiogenic embolism, and BP fluctuations have different effects on ischemic stroke caused by different mechanisms (35), our research will be more targeted. In addition, 33–55% of ischemic stroke in Chinese is caused by intracranial arterial stenosis or occlusion (IASO) (35). Therefore, IASO should be taken into consideration when studying the relationship between BPV and ischemic stroke outcome. In our study, we evaluated intracranial arteries by 3D-TOF-MRA. Due to the limitations of MRA for assessing the degree of stenosis of cerebral arteries, it was difficult to distinguish SIAO from SIAS accurately. Therefore, it would be reasonable to combine SIAO and SIAS into the category of SIASO.

Recent studies have reported that many patients have autonomic dysfunction after ischemic stroke (36–38). BP fluctuation is a manifestation of autonomic dysfunction. During the early stage of ischemic stroke, a rapid increase in BP may cause intracranial pressure to rise and edema or bleeding to occur at the infarction site, which can lead to death or poor functional outcome (25). However, a large decrease in BP may lead to low perfusion in the infarct area; this effect might be much clearer in AIS patients with large-artery stenosis because patients with SIASO are very sensitive to BP fluctuations, especially in the first few hours after AIS (25, 26).

Cerebral autoregulation (CA) is the ability of the brain to regulate its own blood supply, which maintains an adequate and stable cerebral blood flow despite changes in cerebral perfusion pressure. However, CA is impaired after ischemic stroke, and this autoregulation is damaged in patients with SIASO; furthermore, patients with more severe stenosis tend to have more severe dysautoregulation (35). One study reported that CA impairment ipsilateral to the AIS is associated with a larger infarction and poorer clinical outcome compared with patients with unimpaired CA ipsilateral to the AIS (35, 39). With CA impairment, BP fluctuations have an important effect on cerebral blood flow. Moreover, when BPV increases to levels beyond the regulation of CA, it can lead to hypoperfusion or hyperperfusion in the infarction area (35, 39).

In the present study, high SV-24 h after IVT among patients with SIASO increased the risk of poor outcome at 3 months. This finding may help physicians to screen the most high-risk patients with poor prognosis from other high-risk patients at the early stage of AIS, and thus provide more appropriate interventions. Theoretically, since BP fluctuation in early stage of ischemic stroke may lead to hypoperfusion or intracerebral hemorrhage (24), BPV should be associated with END. However, we did not find any correlation between BPV and END. One possible reason is that in our study, there were only 20 patients (18.2%) with END in SIASO group which may not be powerful to detect the difference of BPV between with END and without END groups. Nevertheless, we found a tendency of association between high BPV and the occurrence of END. Besides, previous studies by other researchers have also failed to find a link between short-term outcome (2-week outcome and in-hospital outcome) and BPV during the acute phase of ischemic stroke (11, 39, 40).

The accuracy of BP measurement for patients with AF might be controversial. In theory, irregular ventricular rate in patients with AF may lead to more pronounced fluctuation in BP than in patients with sinus heart rate. However, we didn't exclude patients with AF in our study. Olson et al. analyzed 42 patients with AF in the condition of AF and sinus rhythm, and the result showed they had similar BPV in these two conditions (41). Maria et al. studied 13,827 patients and found no significant difference in BPV between patients with AF and patients with sinus rhythm (42). Besides, Guideline from American Heart Association in 2009 also specified that oscillographic blood pressure monitors were reliable in patients with AF (43).

Commonly-used BPV parameters consist of SV, SD, and CV. In our study, we found that a high SV-24 h, rather than SD-24 h or CV-24 h, may predict 3-month poor outcome in SIASO group. SD represents the dispersion of values around the mean but does not reflect the effect of the measuring sequence of BP. Since CV is calculated from SD, it does not reflect the aforementioned effect, either. SV estimates the variation in successive measurements and thus takes the sequence into account (44). Therefore, SV is more commonly used in studies because it better reflects the time-series variability of BP. In contrast, SD and CV couldn't reflect the temporal changes in the data, which can result in the same SD or CV in individuals with different clinical characteristics (45).

In our study, SV-24 h was higher in patients who were older and more likely to have hypertension or AF. However, an interaction term analysis was performed (Supplementary Table 7) and it demonstrated that SV-24 h was a risk factor of poor outcome independent of age and hypertension, which was similar to a previous review (46).

There were several strengths to our study. First, BPV was calculated by recording hourly BP within 24 h after thrombolysis, which was far more frequently than in other studies and led to more accurate BPV results [11, 14]. Second, to the best of our knowledge, ours was one of the few studies to have focused on AIS patients after IVT and consider the effects of SIASO on prognosis. However, there were also several limitations in our study. First, the sample size was relatively small. Second, our study was a retrospective study. Third, magnetic resonance angiography was used to evaluate arterial stenosis in this study; however, this method is not as accurate as digital subtraction angiography for evaluating arterial stenosis and might overestimate SIASO. Forth, only four stroke patients received EVT during our research from 2017 to 2019 as we were still developing emergent endovascular therapy in our hospital, thus the finding of our study may be limited. Lastly, intermittent cuff measurement was used to monitor BP in the present study, while continuous cuff monitoring or invasive arterial BP are more accurate.

## Conclusion

Systolic SV had a negative relationship with 3-month outcome in AIS patients with SIASO who were treated with IVT, which indicates that BPV may affect the outcome of AIS. Further multi-center prospective studies with larger sample sizes are warranted.

## Data Availability Statement

The raw data supporting the conclusions of this article will be made available by the authors, without undue reservation.

## Ethics Statement

The studies involving human participants were reviewed and approved by the Ethics Committee of Dongguan People's Hospital (approval number: KYKT2018-002). Written informed consent for participation was not required for this study in accordance with the national legislation and the institutional requirements.

## Author Contributions

Y-KC and Y-LL designed the work and analyzed the data. M-XY, W-CZ, D-HQ, W-CZ, and H-PY collected the clinical data. M-XY and Y-LL wrote the manuscript. M-XY and J-HZ wrote the diagrams. All authors read and approved the final manuscript, contributed toward data analysis, drafted and revised the paper, and agreed to be accountable for all aspects of the work.

## Funding

This study was funded by the Guangdong Sci-Tech Commissioner Project, Grant/Award (Number: 20201800500572). The funding body had no role in the design of the study, collection, analysis, and interpretation of the data, and in writing of the manuscript.

## Conflict of Interest

The authors declare that the research was conducted in the absence of any commercial or financial relationships that could be construed as a potential conflict of interest.

## Publisher's Note

All claims expressed in this article are solely those of the authors and do not necessarily represent those of their affiliated organizations, or those of the publisher, the editors and the reviewers. Any product that may be evaluated in this article, or claim that may be made by its manufacturer, is not guaranteed or endorsed by the publisher.
