# Supplementary material for: Effects of Early-Stage Blood Pressure Variability on the Functional Outcome in Acute Ischemic Stroke Patients With Symptomatic Intracranial Artery Stenosis or Occlusion Receiving Intravenous Thrombolysis
Source: Front Neurol. 2022 Mar 8;13:823494. doi: 10.3389/fneur.2022.823494 (PMC8957087; doi:10.3389/fneur.2022.823494)
Supplement: Supplementary file 1 [file Data_Sheet_1.PDF]

Supplemental material

Table S1 | Risk factors of the 3-month poor outcome and END in univariable analysis of non-SIASO group

	Poor outcome (mRS=2-6) (n=71)	Favorable outcome (mRS=0-1) (n=158)	t/X^2/z Value	*P* Value	With END (n=22)	Without END (n=207)	t/X^2/z Value	*P* Value
Age[a] (years)	63.9(11.7)	60.9(12.0)	1.757	**0.008**	59.9(8.4)	62.00(12.3)	0.795	0.427
Men[b] , n (%)	45(63.4%)	117(74.1%)	2.695	0.101	18(81.8%)	144(69.6%)	1.443	0.325
Hypertension[b] , n (%)	53(74.6%)	113(71.5%)	0.240	0.624	16(85.0%)	150(62.2%)	0.001	0.979
Use antihypertensive therapy before IVT[b], n (%)	9(12.7%)	16(10.1%)	0.185	0.667	2(9.1%)	23(11.1%)	0.072	1.000
Diabetes mellitus[b], n (%)	18(25.4%)	44(27.8%)	0.155	0.694	6(27.3%)	56(27.1%)	0.000	0.982
History of hypercholesterolemia[b], n (%)	28(39.4%)	63(49.9%)	0.010	0.921	11(50.0%)	80(38.6%)	1.033	0.309
Smokers/ex-smokers[b], n (%)	21(29.6%)	57(36.1%)	0.921	0.337	8(36.4%)	70(33.8%)	0.057	0.811
AF[b], n (%)	24(33.8%)	26(16.5%)	8.637	**0.003**	2(9.1%)	48(23.2%)	2.316	0.176
History of coronary heart disease[b], n (%)	7(9.9%)	12(7.6%)	0.330	0.566	3(13.6%)	16(7.7%)	0.912	0.405
Previous stroke[b], n (%)	12(16.9%)	21(13.3%)	0.518	0.472	3(13.6%)	33(14.5%)	0.012	1.000
Systolic blood pressure on admission[a]	156.4(25.0)	159.2(25.0)	-0.773	0.440	161.4(30.4)	158.0(24.3)	-0.615	0.539
OTT[a] (minutes)	210(45.1)	189.3(60.1)	2.860	**0.005**	200.0(57.4)	195.3(56.6)	-0.340	0.734

NIHSS on admission[c] (IQR, 25-75)	8.0(5.0-12.0)	4.0(3.0-8.0)	-5.626	**<0.001**	6(4.0-8.0)	5(4.0-9.0)	-0.378	0.706
SV-24h[a]	12.3(4.7)	12.7(5.9)	-0.396	0.693	11.8(4.2)	12.6(5.7)	0.643	0.521
SD-24h[a]	11.5(3.5)	12.1(3.2)	-1.189	0.236	11.0(2.4)	12.0(3.4)	1.274	0.204
CV-24h[a]	8.1(2.5)	8.8(2.4)	-1.853	0.064	7.8(2.1)	8.7(2.5)	1.558	0.121

Note: [a]Mean(SD) ,t-test; [b]n (%), chi-square test; [c]Mann–Whiteny U test.

mRS, modified Rankin Scale; NIHSS, National Institutes of Health Stroke Scale; OTT, Onset to Treatment Time; END, Early Neurological Deterioration; AF, atrial fibrillation; SV-24h, successive variability of systolic blood pressure within the first 24 hours after IVT; SD-24h, standard deviation of systolic blood pressure within the first 24 hours after IVT; CV-24h, coefficient of systolic blood pressure variation within the first 24 hours after IVT; SIASO, Symptomatic Intracranial Artery Stenosis or Occlusion

Table S2 | Multivariate logistic regression of risk factors for 3-month poor outcome in non-SAISO group

**Variable**	**3-month**		
	β	***OR*（95% *CI*）**	***P* value**
Age	0.016	1.016 (0.988-1.045)	0.256
NIHSS on admission	0.204	1.226 (1.132-1.327)	**<0.001**
AF	0.755	2.129 (1.015-4.465)	**0.046**
OTT	0.011	1.011 (1.004-1.017)	**0.001**

NIHSS, National Institutes of Health Stroke Scale; AF, atrial fibrillation; OTT, Onset to Treatment Time; SIASO, Symptomatic Intracranial Artery Stenosis or Occlusion

TABLE S3 | Multivariate logistic regression of SV-24h for 3-month poor outcome in SAISO group (An interactional analysis of age, AF, hypertension and SV-24h).

Variable	3-month		
	β	***OR*（95% *CI*）**	***P* value**
Age	0.045	1.046 (1.009-1.085)	**0.015**
Hypertension	-1.298	0.273 (0.004-16.644)	0.536
AF	-2.506	0.082 (0.000-122.915)	0.502
SV-24h	0.150	1.161 (1.031-1.308)	**0.014**
SV-24h by age	-0.004	0.996 (0.986-1.006)	0.389
SV-24h by hypertension	0.011	1.011 (0.941-1.087)	0.760
SV-24h by AF	0.057	1.059 (0.959-1.168)	0.257

AF, atrial fibrillation; SV-24h, successive variability of systolic blood pressure within the first 24 hours after IVT; SIASO, symptomatic intracranial artery stenosis or occlusion

TABLE S4 | Interaction effects of BPV and SIASO on the 3-month poor outcome in all patients with IVT.

Variable	3-month		
	β	***OR*（95% *CI*）**	***P* value**
Age	0.025	1.025 (1.004-1.047)	**0.021**
NIHSS on admission	0.189	1.208 (1.137-1.283)	**<0.001**
AF	0.433	1.543 (0.796-2.989)	0.199
SV-24h	-0.008	0.992 (0.937-1.050)	0.774
SIASO	-0.947	0.388 (0.067-2.247)	0.291
SV-24h by SIASO	0.099	1.104 (1.060-1.149)	**<0.001**

AF, atrial fibrillation; NIHSS, National Institutes of Health Stroke Scale; SV-24h, successive variability of systolic blood pressure within the first 24 hours after IVT; SIASO, symptomatic intracranial artery stenosis or occlusion

TABLE S5 | BPV patterns of the 3-month poor outcome in univariable analysis of SIASO group.

	Poor outcome (mRS=2-6) (n=73)	Favorable outcome (mRS=0-1) (n=37)	t value	*P* Value
SV-6h[a]	15.1(6.8)	12.1(4.9)	-2.620	**0.010**
SD-6h[a]	11.0(5.0)	9.4(3.7)	-1.810	0.073
CV-6h[a]	7.6(3.1)	6.7(2.6)	-1.511	0.134
SV-12h[a]	14.2(4.7)	11.5(4.0)	-3.115	**0.003**
SD-12h[a]	11.7(4.1)	10.0(3.7)	-2.289	**0.025**
CV-12h[a]	8.2(2.8)	7.3(2.8)	-1.642	0.103

Note: [a]Mean (SD), t-test.

SV-6h, successive variability of systolic blood pressure within the first 6 hours after IVT; SD-6h, standard deviation of systolic blood pressure within the first 6 hours after IVT; CV-6h, coefficient of systolic blood pressure variation within the first 6 hours after IVT; SV-12h, successive variability of systolic blood pressure within the first 12 hours after IVT; SD-12h, standard deviation of systolic blood pressure within the first 12 hours after IVT; CV-12h, coefficient of systolic blood pressure variation within the first 12 hours after IVT; SIASO, symptomatic intracranial artery stenosis or occlusion

TABLE S6 | Multivariate logistic regression of BPV within the first 6 and 12 hours for 3-month poor outcome in SAISO group.

**Variable**	**3-month**		
	β	***OR*（95% *CI*）**	***P* value**
**Model 1**			
Age	0.045	1.046 (1.007-1.087)	**0.020**
NIHSS on admission	0.178	1.195 (1.079-1.322)	**0.001**

AF	0.488	1.628 (0.376-7.057)	0.515
SV-6h	0.069	1.071 (0.980-1.171)	0.130
**Model 2**			
Age	0.039	1.040 (1.001-1.081)	**0.047**
NIHSS on admission	0.176	1.192 (1.074-1.324)	**0.001**
AF	0.511	1.667 (0.385-7.216)	0.494
SD-12h	-0.005	1.035 (0.994-1.079)	0.960
SV-12h	0.116	1.123 (0.992-1.272)	0.066

AF, atrial fibrillation; NIHSS, National Institutes of Health Stroke Scale; SV-6h, successive variability of systolic blood pressure within the first 6 hours after IVT; SV-12h, successive variability of systolic blood pressure within the first 12 hours after IVT; SD-12h, standard deviation of systolic blood pressure within the first 12 hours after IVT; SIASO, symptomatic intracranial artery stenosis or occlusion

TABLE S7 | Univariable analysis of BPV patterns in patients with HT or sHT in SIASO group.

	**With HT (n=33)**	**Without HT (n=77)**	**t value**	***P* Value**	**With sHT (n=6)**	**Without sHT (n=104)**	**t value**	***P* Value**
SV-24h[a]	13.6(3.7)	13.6(4.3)	-0.030	0.977	15.3(3.1)	13.5(4.2)	-1.061	0.291
SD-24h[a]	11.7(3.2)	12.2(4.0)	0.638	0.525	14.2(3.6)	12.0(3.7)	-1.403	0.164
CV-24h[a]	8.4(2.1)	8.7(2.7)	0.402	0.688	9.9(2.5)	8.5(2.5)	-1.313	0.192

Note: [a]Mean (SD), t-test.

SV-24h, successive variability of systolic blood pressure within the first 24 hours after IVT; SD-24h, standard deviation of systolic blood pressure within the first 24 hours after IVT; CV-24h, coefficient of systolic blood pressure variation within the first 24 hours after IVT; HT, hemorrhagic transformation; sHT, symptomatic hemorrhagic transformation, SIASO, symptomatic intracranial artery stenosis or occlusion

TABLE S8 | Univariable analysis of BPV patterns in patients with HT or sHT in non-SIASO group.

	**With HT (n=41)**	**Without HT (n=188)**	**t value**	***P* Value**	**With sHT (n=5)**	**Without sHT (n=224)**	**t value**	***P* Value**
SV-24h[a]	12.0(4.7)	12.7(5.8)	0.654	0.514	12.8(7.5)	12.5(5.6)	-0.117	0.907
SD-24h[a]	11.3(3.3)	12.1(3.3)	1.375	0.171	12.3(1.6)	12.0(3.4)	-0.206	0.837
CV-24h[a]	8.3(2.0)	8.7(2.6)	0.870	0.385	8.6(1.4)	8.6(2.5)	-0.022	0.982

Note: [a]Mean (SD), t-test.

SV-24h, successive variability of systolic blood pressure within the first 24 hours after IVT; SD-24h, standard deviation of systolic blood pressure within the first 24 hours after IVT; CV-24h, coefficient of systolic blood pressure variation within the first 24 hours after IVT; HT, hemorrhagic transformation; sHT, symptomatic hemorrhagic transformation; SIASO, symptomatic intracranial artery stenosis or occlusion
